# Prospective evaluation of changes in choroidal vascularity index after half-dose photodynamic therapy versus micropulse laser treatment in chronic central serous chorioretinopathy

**DOI:** 10.1007/s00417-020-04619-6

**Published:** 2020-03-13

**Authors:** Thomas J. van Rijssen, Sumit Randhir Singh, Elon H. C. van Dijk, Mohammed A. Rasheed, Kiran Kumar Vupparaboina, Camiel J. F. Boon, Jay Chhablani

**Affiliations:** 1 Department of Ophthalmology, Leiden University Medical Center, Leiden, the Netherlands; 2 Department of Ophthalmology, L.V. Prasad Eye Institute, Hyderabad, India; 3 Department of Ophthalmology, Academic Medical Center, University of Amsterdam, Amsterdam, the Netherlands; 4 Department of Ophthalmology, University of Pittsburgh, UPMC Eye Center, Pittsburgh, PA USA

**Keywords:** Central serous chorioretinopathy, Choroidal vascularity index, Micropulse laser, Photodynamic therapy

## Abstract

**Purpose:**

To assess whether treatment of chronic central serous chorioretinopathy (cCSC) with photodynamic therapy (PDT) and high-density subthreshold micropulse laser (HSML) results in choroidal vascularity index (CVI) changes that may account for the treatment effect.

**Methods:**

Patients with cCSC were prospectively included and analyzed. Patients received either half-dose PDT or HSML treatment. CVI of the affected and unaffected eye was obtained before treatment, 6 to 8 weeks after treatment, and 7 to 8 months after treatment.

**Results:**

At baseline, 29 eyes (29 patients) were included both in the PDT and in the HSML group. The mean (± standard deviation) CVI change in the HSML group between before PDT and 6 to 8 weeks after PDT was − 0.009 ± 0.032 (*p* = 0.127), whereas this was 0.0025 ± 0.037 (*p* = 0.723) between the visit before PDT and final visit. The patients in the PDT group had a CVI change of − 0.0025 ± 0.037 (*p* = 0.723) between the visit before PDT and first visit after PDT, and a mean CVI change of − 0.013 ± 0.038 (*p* = 0.080) between the visit before PDT and final visit. There was no significant correlation between CVI and BCVA at the measured time points, in both the HSML group (*p* = 0.885), and in the PDT group (*p* = 0.904). Moreover, no significant changes in CVI occurred in the unaffected eye at any time point.

**Conclusions:**

PDT and HSML do not significantly affect CVI, and therefore a CVI change may not be primarily responsible for the treatment effect. The positive treatment effect of both interventions may rely on other mechanisms, such as an effect on choriocapillaris and/or retinal pigment epithelium function.

## Introduction

Central serous chorioretinopathy (CSC) is characterized by the presence of subretinal fluid (SRF), presumably caused by underlying choroidal abnormalities that lead to damage to the retinal pigment epithelium (RPE) [1]. CSC has been associated with multiple risk factors including male gender, type A personality, steroid use, Cushing disease, and genetic predisposition [2–5]. For this chorioretinal disease, variable practice patterns exist among clinicians [6]. Photodynamic therapy (PDT) [7–10], high-density subthreshold micropulse laser (HSML) [11–14], and mineralocorticoid antagonist-like eplerenone have been used to treat chronic CSC [4, 15–17]. However, treatment options are not uniformly successful with variable and suboptimal outcomes, and different thoughts about the desired effect of treatment [18, 19]. These controversies regarding the first-line treatment modality for chronic CSC exist till date due to lack of level I evidence [19].

The half-dose photodynamic therapy versus high-density subthreshold micropulse laser treatment in patients with chronic central serous chorioretinopathy (PLACE) study was a prospective, randomized controlled trial conducted in 5 centers across Europe [20, 21]. The superiority study compared half-dose photodynamic therapy with high-density subthreshold micropulse laser treatment. The primary outcome measure being studied was complete resolution of SRF as measured on optical coherence tomography (OCT) at the first evaluation visit at 6 to 8 weeks after first treatment. Moreover, complete resolution of SRF on OCT at final follow-up at 7 to 8 months after first treatment and best-corrected visual acuity (BCVA), retinal sensitivity on microperimetry, and visual functioning through a validated questionnaire at the first evaluation visit were the secondary study outcome parameters [21]. According to the results of the PLACE trial, half-dose PDT is superior to HSML treatment both in terms of anatomical and functional outcome [20]. However, the analysis of choroidal vasculature, including choroidal vascularity index (CVI) was not part of the study.

CVI and lumen/choroid ratio have been described as ratios to assess the luminal area versus total choroidal area [22–25]. The ratio expressed as a percentage usually varies from 64 to 67% in normal individuals and is known to alter in various pathologies including CSC [22–24, 26, 27]. It has been proposed earlier that CVI may act as an indicator of disease activity in CSC [26]. This was based on the assumption that choroidal vascular hyperpermeability forms the main pathogenetic conduit for onset of CSC, and thus monitoring the changes in CVI over a period of time may be useful to provide information about the disease.

In the present study, we evaluated the changes in CVI after PDT and HSML and analyzed variables that may have a positive effect on changes in BCVA. The changes in CVI during follow-up may help predicting both the response to several treatments and disease progression.

## Methods

The PLACE trial was a randomized controlled trial comparing half-dose PDT with HSML [21]. The subjects included in this study were recruited from 5 centers across Europe from November 22, 2013 to September 15, 2016 [21]. In the present study, we have analyzed a subset of PLACE trial patients originating from 1 of the participating centers (Leiden University Medical Center, Leiden, Netherlands).

### Study design

The details on the study design, patient inclusion and exclusion criteria, interventions involved, and primary and secondary endpoints have been described in a previous publication [21]. In brief, patients were randomized into 2 groups: 1 group received half-dose PDT and the other group received HSML treatment. The same treatment, if deemed required at an evaluation visit at 6 to 8 weeks after the first treatment, could be repeated. The total duration of follow-up for all patients was 7 to 8 months.

The patients included were cases with active, chronic CSC, above 18 years of age with an accumulation of SRF on OCT and/or a subjective vision loss of more than 6 weeks. The patients had SRF that involved the fovea, together with presence of hyperfluorescent hot spots of leakage on fluorescein angiography (FA) and corresponding hyperfluorescent changes on indocyanine green angiography (ICGA). The baseline evaluation included fundus photography, OCT, fundus autofluorescence (FAF), FA, and ICGA, and the images were sent to a central reading center for assessment of the eligibility for inclusion.

Fundus photographs were obtained with a fundus camera from Topcon Medical Systems (Oakland, NJ, USA). OCT, FAF, FA, and ICGA were obtained with Spectralis HRA + OCT; (Heidelberg Engineering, Heidelberg, Germany). The subfoveal choroidal thickness (SFCT) was obtained by measuring the distance between Bruch’s membrane and the outer scleral border or spectral-domain (SD) OCT.

### Choroidal vascularity index calculation

**Fig. 1:**
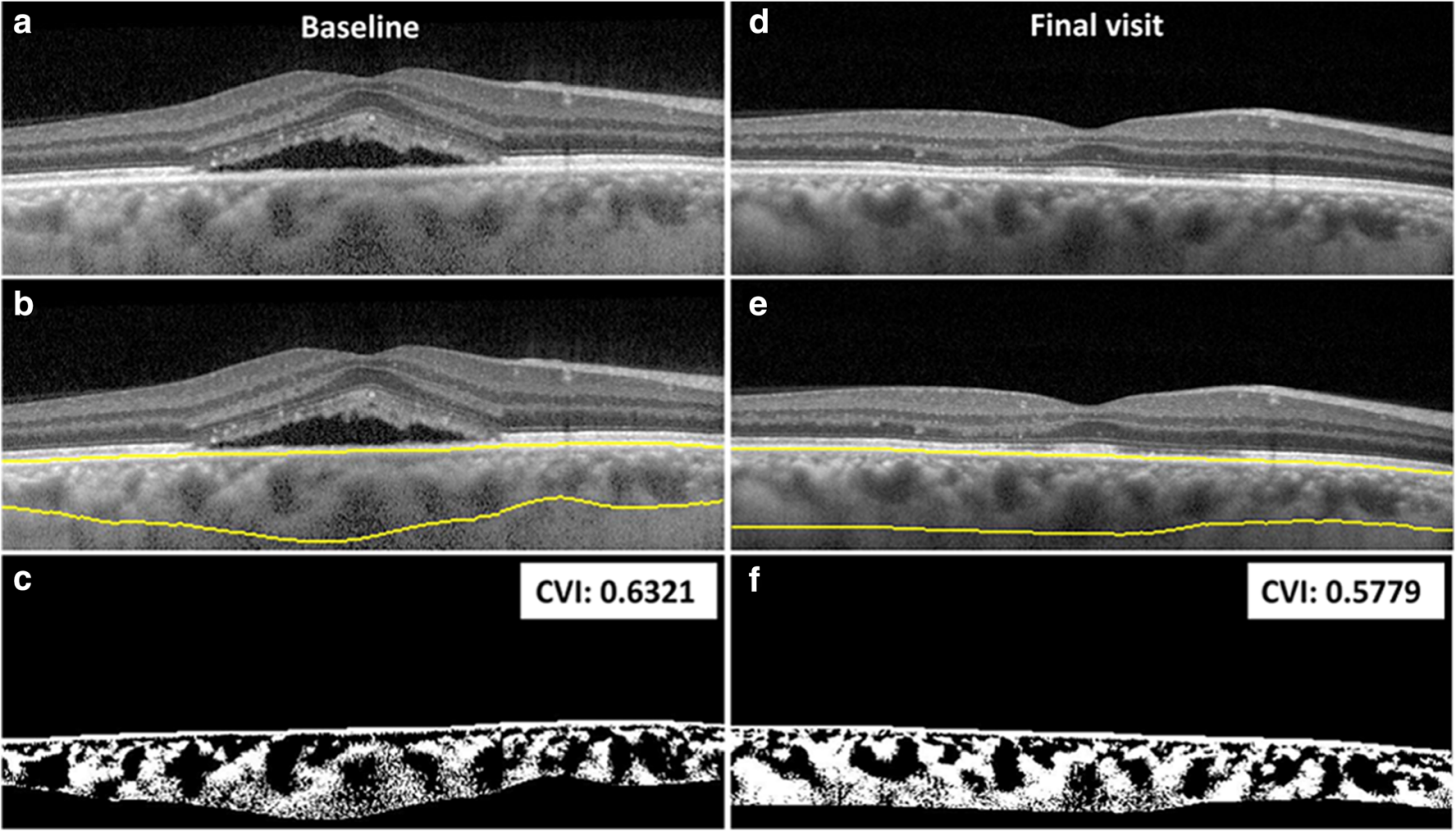
Optical coherence tomography imaging of the right eye (**a**–**f**) of a 41-year-old male patient affected with chronic central serous chorioretinopathy and treated with HSML. Baseline imaging (**a–c**) and final visit imaging obtained 7–8 months later (**d**–**f**), including the yellow segmentation lines (**b**, **e**) for the calculation of the CVI are depicted. The CVI is 0.6321 at baseline and changed to 0.5779 at final visit (change was not significant). CVI, choroidal vascularity index; HSML, high-density subthreshold micropulse laser

**Fig. 2:**
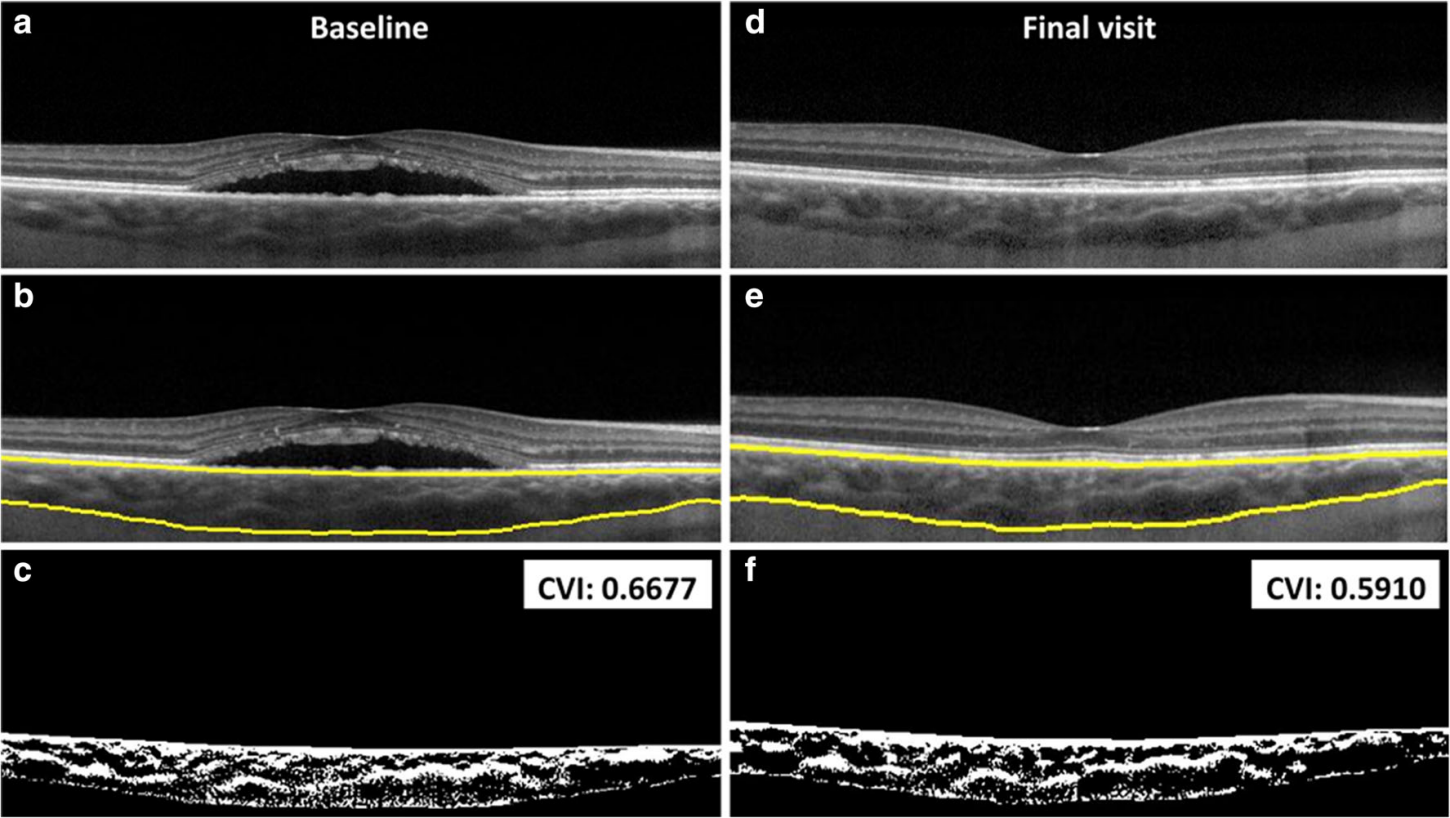
Optical coherence tomography imaging of the right eye (**a**–**f**) of a 50-year-old male patient affected with chronic central serous chorioretinopathy and treated with PDT. Baseline imaging (**a**–**c**) and final visit imaging obtained 7–8 months later (**d–f**), including the yellow segmentation lines (**b**, **e**) for the calculation of the CVI are depicted. The CVI is 0.6677 at baseline and changed to 0.5910 at final visit. CVI, choroidal vascularity index; PDT, photodynamic therapy

The CVI calculation was done using a previously reported algorithm [24, 28]. Briefly, choroidal stroma and vessel area analysis involved (I) automated binarization of a high-definition horizontal 6 mm OCT B-scan and (II) automated segmentation of the binarized choroid layer as reported previously [28]. The task of automated binarization involved (a) preprocessing, (b) exponential and non-linear enhancement, and (c) thresholding (Fig. 1b; Fig. 2b). The inter-observer variability of this CVI calculation has previously been reported to be 98% before shadow compensation [29]. The total subfoveal choroidal area and luminal area measured in a previous study both had an intraclass correlation coefficient of 94% [22]. These previous findings indicate a robust inter-observer variability of the CVI calculation.

### Statistical methods

The data were compiled, tabulated, and analyzed using Statistical Package for the Social Sciences software (version 22; IBM Corp., New York, NY). Linear mixed model analyses were used to study the changes in BCVA, CVI, and SFCT over time and the correlation between CVI and BCVA in time. In the mixed model, BCVA was used as the dependent variable, time was used as a factor, and CVI was used as a covariate. *p* values ≤ 0.05 were considered statistically significant. A Pearson correlation test was performed in order to analyze the correlation between the CVI and SFCT.

## Results

A total of 58 eyes of 58 patients were included in the analysis: 29 eyes each in the PDT and HSML group. OCT imaging and CVI measurements are shown from a patient treated with HSML and a patient treated with PDT in Fig. 1a–f and Fig. 2a–f, respectively. The study group mainly comprised males (86.2%), and the mean (± standard deviation (SD)) age at presentation was 47.9 ± 7.8 years. The baseline demographics of both the HSML and PDT groups are depicted in Table 1. The SFCT could not be measured in 3 patients at baseline and at an evaluation visit at 6 to 8 weeks after the first treatment. At final visit, the SFCT was unavailable in 6 patients. This was due to insufficient imaging quality of the SD-OCT scan.

**Table 1:** Baseline characteristics of chronic central serous chorioretinopathy patients treated with either half-dose photodynamic therapy or high-density subthreshold micropulse laser

		HSML		Half-dose PDT		*p* value
		Count	Mean (SD)	Count	Mean (SD)	
Sex	Male	26 (90%)		24 (83%)		0.706
	Female	3 (10%)		5 (17%)		
Age (in years)			48 ± 7.6		47 ± 8.1	0.622
BCVA at baseline (in ETDRS letters)			76 ± 10.3		78 ± 8.2	0.510
CVI at baseline			0.61 ± 0.031		0.60 ± 0.037	0.344
Choroidal thickness at baseline (μm)			417 ± 116		438 ± 112	0.496

*BCVA* best-corrected visual acuity, *CVI* choroidal vascularity index, *ETDRS* Early Treatment of Diabetic Retinopathy Study, *HSML* high-density subthreshold micropulse laser, *PDT* photodynamic therapy, *SD* standard deviation

### Changes in CVI and SFCT

At baseline, the mean (± SD) CVI for the total sample size was 0.6034 ± 0.0346, and at the last follow-up, CVI was 0.6025 ± 0.0310 (Δ − 0.09). The change in CVI, when comparing CVI before and after treatment for the 2 combined groups, was not significant (*p* = 0.452). A subanalysis involving PDT or HSML was also performed. The mean (± SD) change in CVI in the HSML group between baseline and evaluation visit was − 0.0093 ± 0.032, which was not statistically significant (*p* = 0.127). In contrast, the patients in the PDT-treated group (*n* = 29 eyes) had a minimal increase in CVI (+ 0.0025 ± 0.037), which was not statistically significant (*p* = 0.723). There was a mean change in CVI of + 0.011 between baseline and final visit in the HSML group (*p* = 0.118), whereas a mean change in CVI of − 0.013 between baseline and final visit (*p* = 0.080) was found in the PDT-treated group. In the HSML group, the mean SFCT was 417 ± 116 μm at baseline, 422 ± 117 μm at evaluation visit (*p* = 0.865), and 394 ± 112 μm at final visit (*p* = 0.464, compared to baseline). In the half-dose PDT group, mean SFCT was 438 ± 114 μm at baseline, 371 ± 129 μm at evaluation visit (*p* = 0.039), and 342 ± 110 μm at final visit (*p* = 0.004, compared to baseline).

### Correlation of CVI and SFCT

In the HSML group, the Pearson correlation between the CVI change between baseline and final visit, and the SFCT change between baseline and final visit was 0.011 (*p* = 0.957). This Pearson correlation was 0.138 in the PDT group (*p* = 0.500).

### Correlation of CVI with BCVA

A mixed model was computed to analyze the correlation between change in CVI and change in BCVA for the total sample size, and for the patients that were treated with either HSML or PDT treatment separately. When evaluating the total sample size, CVI was not correlated with BCVA (estimate = 18.90, standard error = 26.79, *p* = 0.483). When subanalyzing both treatment groups, CVI did not have a significant effect on BCVA in both the HSML group (estimate = 5.87, standard error = 40.12, *p* = 0.885) and in the PDT group (estimate = − 4.73, standard error = 39.06, *p* = 0.904).

### Fellow eye

Mean Early Treatment of Diabetic Retinopathy Study (ETDRS) BCVA in the HSML group was 88.8 ± 7.6 at baseline, 88.9 ± 7.5 at evaluation visit, and 88.8 ± 9.3 at final visit (*p* > 0.999, linear mixed model). In the half-dose PDT group, mean ETDRS BCVA was 87.9 ± 7.6 at baseline, 88.3 ± 7.5 at evaluation visit 1, and 88.6 ± 8.6 at final visit (*p* = 0.951, linear mixed model).

Mean CVI in the HSML group was 0.597 ± 0.040 at baseline, 0.605 ± 0.035 at evaluation visit, and 0.596 ± 0.037 at final visit (*p* = 0.586, linear mixed model). The mean CVI in the half-dose PDT group was 0.610 ± 0.036 at baseline, 0.589 ± 0.085 at evaluation visit, and 0.605 ± 0.036 at final visit (*p* = 0.350, linear mixed model).

In the HSML group, the mean SFCT was 401 ± 143 μm at baseline, which decreased to 389 ± 133 μm at evaluation visit, and to 377 ± 119 μm at final visit (*p* = 0.799, linear mixed model). In the half-dose PDT group, mean SFCT was 375 ± 136 μm at baseline, 378 ± 143 μm at evaluation visit, and 370 ± 150 μm at final visit (*p* = 0.977, linear mixed model).

## Discussion

CSC is a chorioretinal disease characterized by increased choroidal thickness, hyperpermeability, and congestion of choroidal vessels [4, 5, 30]. Various studies have assessed these choroidal characteristics to assess and predict disease course and response to treatment [26, 31, 32]. CVI has recently been introduced as a marker of disease activity and has been reported to be increased in acute, chronic CSC (cCSC), and resolved CSC cases as compared to healthy controls [26].

In the first large multicenter randomized controlled trial (PLACE trial) on cCSC, patients that were treated with half-dose PDT were found to have a significantly higher percentage of complete resolution of SRF as compared to HSML [20]. After all, PDT is thought to primarily target the choroid, and after a short-term initial choroidal thickness increase, a reduction in choroidal thickness was found after PDT for cCSC, which may account for a post-treatment difference in CVI compared to HSML treatment [33]. In contrast to PDT, HSML is thought to primarily act through an effect on RPE function, although the precise mechanisms are unclear [11].

Somewhat surprisingly, we did not find a statistically significant reduction in CVI after either half-dose PDT or HSML in this prospective study. The superior anatomical and functional outcome of half-dose PDT in comparison to HSML treatment may therefore not be correlated with changes in CVI. Park et al. reported a significant decrease in CVI after half or full-dose PDT, and a significant increase in CVI after half-dose-half-fluence PDT [34]. This is in contrast with our study, since no significant changes in CVI were observed after half-dose PDT. In the study of Park et al., both acute CSC and cCSC patients were included, and the follow-up time within this study was only 3 months. Our study only included cCSC patients and had a follow-up of 8 months. These differences between the study of Park et al. and our study may explain the discrepancies in study findings, also given the possible differences between several entities within the pachychoroid/CSC spectrum [35]. The treatment effect of PDT may rather be a consequence of vascular remodeling of the choriocapillaris and larger choroidal vessels at the level of the vessel walls and their permeability, while their luminal density relative to the total choroidal area (calculated as CVI) may not be significantly affected. However, the choroidal thickness often decreases after half-dose PDT [36]. In the HSML group, we did not find an association between CVI change and the change in BCVA after treatment. HSML presumably may have an effect primarily on the RPE and does not directly affect choroidal vasculature.

In the untreated fellow eye, no significant changes in BCVA, CVI, and SFCT were observed, which may indicate that these parameters do not significantly change within a natural clinical course of 8 months. Effects of treatment on the contralateral eye are presumably negligible due to the local application of both treatments. Limitations of the current study were the relatively small sample size, and the relatively limited follow-up of 7 to 8 months. In addition, CVI is a relatively new endpoint and therefore limited studies are available. To assess whether an improvement in BCVA induced by either PDT or HSML may have a relationship with changes in CVI needs further validation with a larger sample size and longer follow-up.

In conclusion, both half-dose PDT and HSML treatment do not significantly affect CVI in chronic CSC. The treatment effects of both treatment modalities may therefore rely on other mechanisms, such as a reduction of choroidal hyperpermeability and leakage.
